# The Relationship between Anogenital Distance, Fatherhood, and Fertility in Adult Men

**DOI:** 10.1371/journal.pone.0018973

**Published:** 2011-05-11

**Authors:** Michael L. Eisenberg, Michael H. Hsieh, Rustin Chanc Walters, Ross Krasnow, Larry I. Lipshultz

**Affiliations:** 1 Division of Male Reproductive Medicine and Surgery, Scott Department of Urology, Baylor College of Medicine, Houston, Texas, United States of America; 2 Department of Urology, Stanford University School of Medicine, Palo Alto, California, United States of America; University of Muenster, Germany

## Abstract

**Background:**

Anogenital distance (AGD), a sexually dimorphic measure of genital development, is a marker for endocrine disruption in animal studies and may be shorter in infant males with genital anomalies. Given the correlation between anogenital distance and genital development, we sought to determine if anogenital distance varied in fertile compared to infertile adult men.

**Methods:**

A cross sectional study of consecutive men being evaluated for infertility and men with proven fertility was recruited from an andrology clinic. Anogenital distance (the distance from the posterior aspect of the scrotum to the anal verge) and penile length (PL) were measured using digital calipers. ANOVA and linear regression were used to determine correlations between AGD, fatherhood status, and semen analysis parameters (sperm density, motility, and total motile sperm count).

**Findings:**

A total of 117 infertile men (mean age: 35.3±17.4) and 56 fertile men (mean age: 44.8±9.7) were recruited. The infertile men possessed significantly shorter mean AGD and PL compared to the fertile controls (AGD: 31.8 vs 44.6 mm, PL: 107.1 vs 119.5 mm, p<0.01). The difference in AGD persisted even after accounting for ethnic and anthropomorphic differences. In addition to fatherhood, on both unadjusted and adjusted linear regression, AGD was significantly correlated with sperm density and total motile sperm count. After adjusting for demographic and reproductive variables, for each 1 cm increase in a man's AGD, the sperm density increases by 4.3 million sperm per mL (95% CI 0.53, 8.09, p = 0.03) and the total motile sperm count increases by 6.0 million sperm (95% CI 1.34, 10.58, p = 0.01). On adjusted analyses, no correlation was seen between penile length and semen parameters.

**Conclusion:**

A longer anogenital distance is associated with fatherhood and may predict normal male reproductive potential. Thus, AGD may provide a novel metric to assess reproductive potential in men.

## Introduction

Over the past half century there has been a reported decline in semen quality and male births with an increased rate in male genital abnormalities and testis cancers [1], [2], [3]. While the phenomenon and etiology is uncertain, several groups postulate an environmental factor which disrupts normal endocrine signaling leading to abnormal androgen action and altered testicular development [3], [4]. Investigators have used the anogenital distance (AGD) as a measure of genital development and androgen status in both experimental animals and humans in an attempt to gauge reproductive toxicities.

As males have longer anogenital lengths than females, AGD was initially used to sex animals [5], [6], [7]. Human studies have also validated such findings in infants and toddlers demonstrating that boys have longer perineal lengths than girls [8], [9], [10], [11]. Investigators have used such a gender discrepancy to show that agents which disrupt androgen signaling can lead to abnormal genital lengths in animal models [12], [13]. Scott et al showed that in addition to reduced anogenital lengths, rodents exposed to certain phthalates, which are known to suppress fetal androgen levels, had altered testicular size and Sertoli cell function [12].

Swan et al demonstrated that male infants of mothers exposed to increasing levels of known endocrine disruptors had shorter anogenital lengths suggesting an impairment of *in utero* male genital development [14], Moreover, Hsieh et al studied young boys undergoing elective urological surgery and showed that boys with more severe genital anomalies (i.e. hypospadias and cryptorchidism) had significantly shorter anogenital lengths compared to boys with no genital anomalies [6].

To date, there are no studies which explore anogenital distance in adult males. Moreover, as the anogenital distance has been shown to vary based on the integrity of androgen pathways, it is possible that a shorter anogenital distance may signal impaired testicular function in men. Indeed, rodent studies have established critical gestational windows where genital development (i.e. penile length, AGD, testis weight) can be irreparably altered by exposure to endocrine disruptors [15], [16]. As testicular and penile development and function are related to anogenital distance in rodents, we sought to determine if human testicular function is related to anogenital length.

## Methods

### Study population

After obtaining Institutional Review Board approval from Baylor College of Medicine, eligible patients were recruited from a urology clinic specializing in reproductive medicine beginning in August 2010. Patients evaluated for male infertility (primary or secondary) over 18 years of age were eligible. Men with a history of orchiectomy, testicular torsion, or prior malignancy were excluded. From the same practice, a fertile control group (i.e. history of prior paternity) was assembled. Such men were undergoing vasectomy, vasectomy reversal, sperm retrieval after vasectomy, or office evaluation of hypogonadism or erectile dysfunction. All men provided written consent for participation. Age, self-reported race, height, and weight were recorded.

### Genital measurements

**Figure 1:**
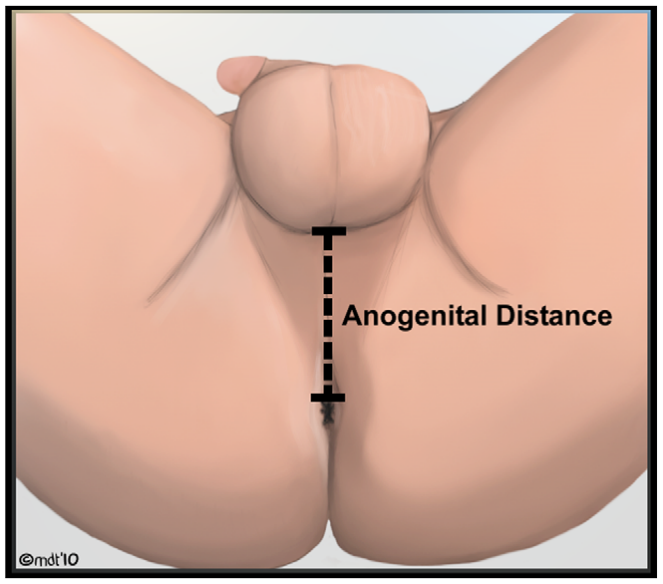
Anogenital distance as measured with men in supine, frog-legged position.

In the supine, frog-legged position with the legs abducted allowing the soles of the feet to meet, the distance from the posterior aspect of the scrotum to the anal verge was measured using a digital caliper (Neiko USA, Model No. 01407A) (Figure 1). The soles of the feet were between 12 to 18 inches from the buttocks for all measurements. The anal verge was marked as the anterior most point where the anus begins. The posterior aspect of the scrotum was the point where the rugated scrotal skin meets the perineum. It is important to note that others have defined the anogenital distance (AGD) from the anus to the anterior base of the penis and the distance from the posterior scrotum to the anus (as was measured in this study) as the anoscrotal distance (ASD) [6], [11], [14]. Given the age of the patients measured, the posterior scrotum was measured as the anterior border as it was felt to be a more comfortable, reliable, and reproducible measure.

From the same position, the stretched penile length (PL) was measured from the base of the dorsal surface of the penis to the tip of the glans. Testicular volume was estimated from the physical examination of one investigator (LIL) at approximately 25 to 27 degrees Celsius.

### Semen analysis

As part of our routine practice, all patients evaluated for infertility have at least two semen analyses performed. The first specimen collected was used for analyses. Several patients not evaluated for infertility also provided semen analyses for other purposes (e.g. sperm banking prior to vasectomy or directed semen donation). Semen analyses from patients who had surgical correction of ductal system abnormalities prior to semen collection were not included in the analysis (e.g. vasectomy, vasectomy reversal, ejaculatory duct obstruction). Thus, for the purposes of the semen data, most of the fertile men with semen data available were men evaluated for secondary infertility. Using WHO guidelines, semen analyses were performed manually on all patients evaluated for infertility within one hour of collection. The volume, density (million per mL), and motility were recorded. Volume, percent motility, and density were multiplied to determine the total motile sperm count.

### Hormone values

Testosterone, LH, and FSH are routinely collected on all patients evaluated for infertility, hypogonadism, or sexual dysfunction. All hormone assays were processed by a single, experienced laboratory (Laboratory for Male Reproductive Research and Testing, Baylor College of Medicine, Houston, Texas). Testosterone, LH, and FSH values were assessed using a one-step competitive binding assay with the Beckman Coulter Access II Immunoassay system (Beckman Coulter, Inc., Brea, California).

### Statistical analysis

Chi-squared tests were used to evaluate the association between categorical variables. ANOVA tests were used to compare continuous variables. The Wilcoxon test was also performed on nonparametrically distributed continuous variables. Pearson correlation coefficients were calculated to assess the relationship of genital measures. Interrater reliability was assessed using the mean standard deviation and Pearson correlation coefficients. Linear regression models were used to determine the relationship between genital measures and semen parameters. Given the nonparametric distribution of the semen parameters and AGD, linear regression models were also run with log transformed variables with no differences in the overall conclusions. AGD, PL, and testis volume were analyzed as continuous values for all analyses. To assess for effect modification by age or ethnicity, stratified analyses were performed with no change in the conclusions. All p values were two sided. Analyses were performed using Stata 10 (StataCorp LP, College Station, Texas).

## Results

In all, 117 men were evaluated for infertility (mean age ± standard deviation: 35.3±17.4), including 97 for primary infertility and 20 for secondary infertility. A total of 12 men were excluded (4 had a prior orchiectomy, 2 had a history of torsion, 3 for previous chemical exposure (e.g. chemotherapy), 2 had inadequate measurements, and 1 had a prior ejaculatory duct resection). An additional 56 fertile controls (mean age ± s.d.: 44.8±9.7) were also recruited. 64.1% of the cohort was white, 12.7% Hispanic, and 12.1% African American. Demographic and hormonal variables are listed in Table 1.

**Table 1:** Demographic, anthropomorphic, hormonal, and reproductive characteristics of the cohort.

		Father				
		No		Yes		
Characteristic		n	Mean (S.D.) or %	n	Mean (S.D.) or %	p
**Age (yrs)**		97	34.3 (6.0)	75	43.6 (10.3)	<0.01
**Height (m)**		95	1.80 (0.08)	74	1.78 (0.08)	0.30
**Weight (kg)**		95	96.0 (24.0)	73	91.8 (18.3)	0.21
**BMI**		95	29.8 (7.3)	73	28.9 (5.7)	0.38
**Testosterone (nmol/L)**		89	10.9 (4.5)	22	11.9 (5.2)	0.36
**FSH (mIU/mL)**		89	8.7 (7.9)	17	5.2 (3.0)	0.08
**LH (mIU/mL)**		84	4.9 (3.2)	17	3.6 (1.5)	0.09
Races*	**White**	61	62.9	49	65.3	0.32
	**African American**	12	12.4	9	12.0	
	**Hispanic**	10	10.3	12	16.0	
	**Asian**	10	10.3	2	2.7	
	**Other**	4	4.1	3	4.0	
**Months Trying**		85	25.2 (22.0)		N.A.	

Comparisons made using ANOVA for continuous variables and Chi-squared for categorical variables with relevant p value displayed.

**Figure 2:**
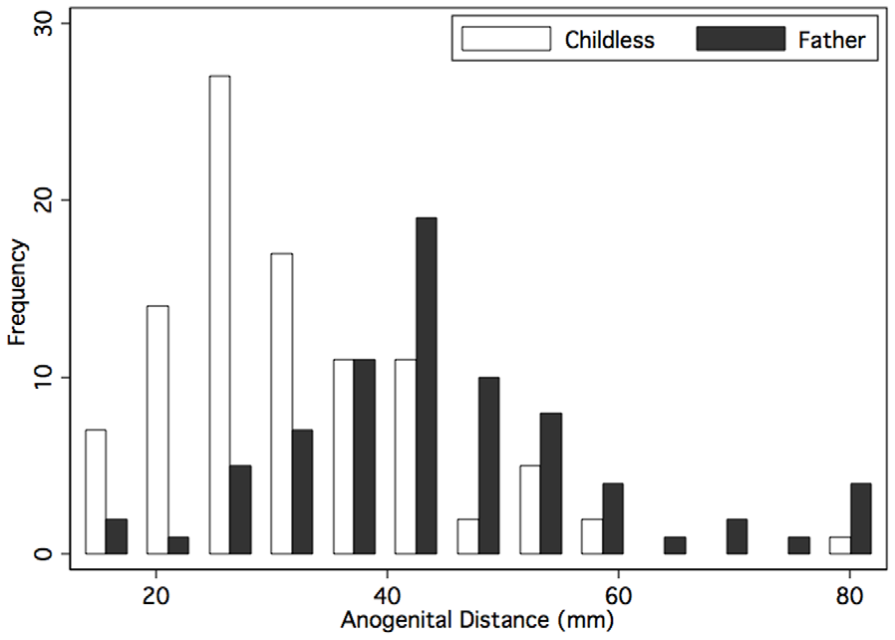
Distribution of anogenital lengths in men that were childless and being evaluated for infertility and men with proven fertility.

**Figure 3:**
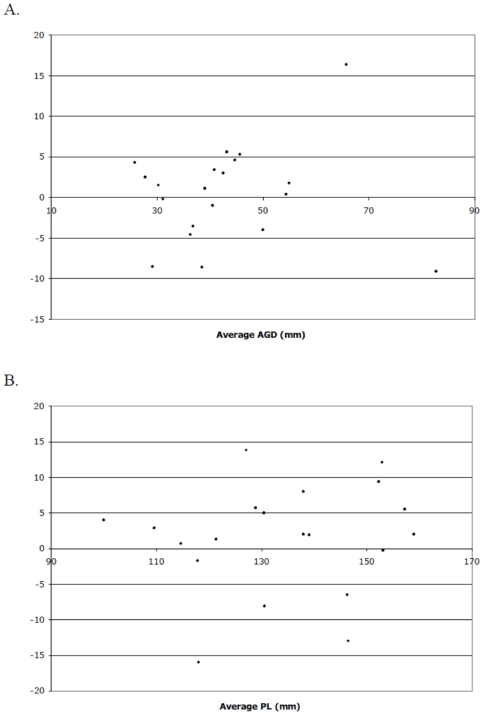
Bland-Altman plot showing the difference in anogenital distance (AGD; Plot A) and penile length (PL, Plot B) measurements as recorded by separate investigators.

Compared to fathers, childless men had significantly shorter AGD (31.8 vs 44.6 mm, p<0.01, Table 2, Figure 2). AGD was measured by 5 investigators (Four authors (MLE, RCW, RK, LIL) and a collaborator). Twenty men had measurements made by two or more investigators. The within-subject standard deviation was 4.1 mm for anogenital distance and 5.4 mm for stretched penile length. There was no evidence for the measurement error being proportional to the magnitude of the measurement (Figure 3). The correlation coefficient was 0.91 for both anogenital distance and penile length measurements.

**Table 2:** Genital measurements and semen characteristics of the cohort.

	Father				
	No		Yes		
Characteristic	n	Mean (S.D.)	n	Mean (S.D.)	p
**AGD (mm)**	97	31.8 (11.3)	75	44.6 (14.1)	<0.01
**Stretched Penile Length (mm)**	95	107.1 (23.0)	74	119.5 (22.7)	<0.01
**Total Testicular Volume (mL)**	97	33.2 (8.0)	75	40.8 (6.7)	<0.01
**Semen Volume**	95	2.7 (1.2)	18	2.7 (1.1)	0.99
**Sperm Density (million/mL)**	95	16.2 (24.0)	18	33.0 (27.9)	<0.01
**Sperm Motility (%)**	95	24.4 (20.2)	18	40.3 (14.4)	<0.01
**Total Motile Sperm Count (millions)**	95	16.8 (30.1)	18	39.3 (45.4)	<0.01

Comparisons made using ANOVA with relevant p value displayed. Wilcoxon test used for sperm density and total motile sperm count.

While a majority of the cohort was white (64%), even when stratified by race, AGD differences between fathers and infertile men remained stable although low sample size limited analysis of some races (Table 3). In addition, infertile men also had shorter stretched penile lengths (107.1 vs 119.5 mm, p<0.01) and total testicular volumes (33.2 vs 40.8 mL, p<0.01) compared to fertile men. In contrast, no significant difference was seen for serum testosterone, LH, or FSH. No significant association was found between BMI, height, or weight and AGD. All genital measurements seemed to be correlated with each other. AGD with penile length (r = 0.20, p<0.01), AGD with total testicular volume (r = 0.31, p<0.01), and penile length with total testicular volume (r = 0.24, p<0.01).

**Figure 4:**
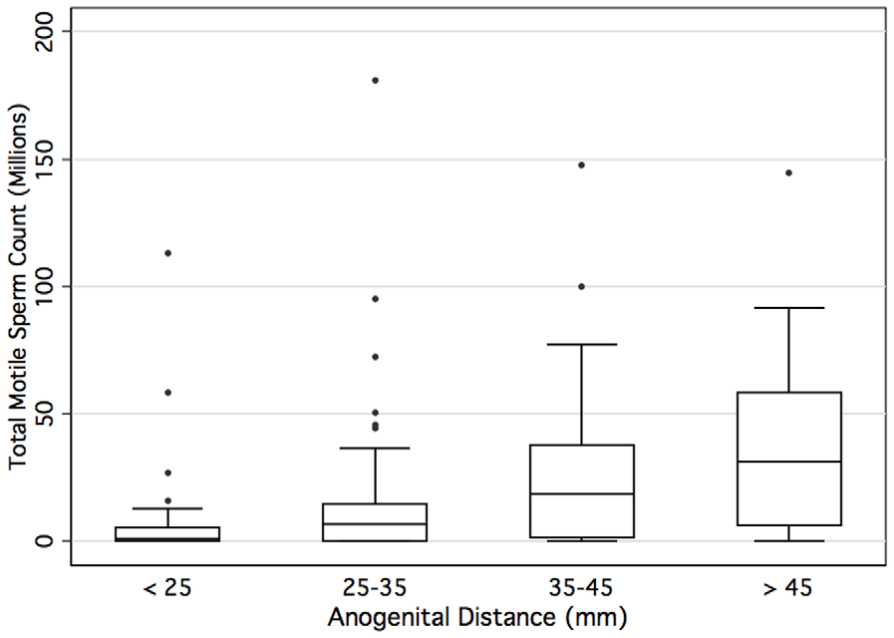
Boxplot showing the interquartile range (IQR) of the total motile sperm count stratified by anogenital lengths. Median value is denoted with horizontal bar. Whiskers designate 1.5× IQR.

While semen volume was similar between fertile and infertile men, sperm density, motility, and total motile sperm count was significantly lower for infertile men (Table 2). Moreover, among all men with semen analysis data, as the AGD lengthens, the total motile sperm count increases (Figure 4).

**Table 3:** Anogenital distance measurements stratified by race and fatherhood.

	Father				
	No		Yes		
	n	Mean (S.D.)	n	Mean (S.D.)	p
**White**	61	33.2 (11.6)	49	41.6 (12.3)	<0.01
**African American**	12	34.1 (9.2)	9	55.4 (17.7)	<0.01
**Hispanic**	10	23.4 (6.7)	12	46.0 (13.1)	<0.01
**Asian**	10	30.9 (9.9)	2	40.1 (2.1)	0.24
**Other**	4	26.2 (17.5)	3	57.3 (22.1)	0.09

Comparisons made using ANOVA with relevant p value displayed.

In both the unadjusted and adjusted models; AGD and testicular volume significantly correlated with total motile sperm count and sperm density. In fact, for each 1 cm increase in AGD, the sperm density increased by 4.3 million sperm per mL (95% CI 0.53, 8.09, p = 0.03) and the total motile sperm count increase by 6.0 million (95% CI 1.34, 10.58, p = 0.01). In contrast, no significant correlation was seen with penile length and sperm count (Table 4).

**Table 4:** Multivariable linear regression model of the relationship between genital measurements and semen parameters.

Semen Parameter	Genital Measurement*	Unadjusted		Adjusted†	
		β (95% CI)	p	β (95% CI)	p
**Semen Volume**	**AGD**	0.12 (−0.06, 0.30)	0.18	0.22 (0.01, 0.42)	0.04
	**Penile Length**	0.04 (−0.05, 0.13)	0.40	−0.03 (−0.15, 0.09)	0.63
	**Testicular Volume**	−0.05 (−0.33, 0.23)	0.73	0.17 (−0.23, 0.57)	0.40
**Sperm Density**	**AGD**	5.55 (1.85, 9.25)	<0.01	4.31 (0.53, 8.09)	0.03
	**Penile Length**	−0.29 (−2.29, 1.71)	0.77	−0.62 (−2.90, 1.66)	0.59
	**Testicular Volume**	12.19 (6.64, 17.74)	<0.01	9.21 (2.05, 16.36)	0.01
**Sperm Motility**	**AGD**	2.89 (−0.15, 5.92)	0.06	2.25 (−0.95, 5.46)	0.17
	**Penile Length**	−0.44 (−2.06, 1.18)	0.59	0.19 (−1.72, 2.11)	0.84
	**Testicular Volume**	4.31 (−0.43, 9.06)	0.07	−0.14 (−6.31, 6.04)	0.97
**Total Motile Sperm Count**	**AGD**	7.16 (2.21, 12.12)	0.01	5.96 (1.34, 10.58)	0.01
	**Penile Length**	0.58 (−2.12, 3.28)	0.67	0.55 (−2.28, 3.38)	0.70
	**Testicular Volume**	14.10 (6.52, 21.67)	<0.01	10.78 (1.94, 19.62)	0.02

*AGD and Penile length in 10 mm increments, Testicular volume in 10 mL increments.

† Adjusted for age, race, FSH, BMI.

## Discussion

The current study demonstrated an association between anogenital distance and fatherhood in a cohort of U.S. adult men evaluated in an andrology practice. In addition, anogenital distance was positively correlated with a man's fertility potential as assessed by sperm production. While differences in genital measurements did exist between some ethnicities, the association with fatherhood remained. To our knowledge, the current study represents the first assessment of anogenital distance in adult men as well as the first examination of the relationship between anogenital distance and a man's fertility.

During sexual development the immature genital precursors migrate ventrally via an androgen mediated pathway [17]. The anogenital distance has been used to sex animals, since males have longer lengths than females [5], [6], [7]. Moreover, human studies in infants have also established that boys have longer perineal lengths than girls [8], [9], [10], [11]. Investigators have used the anogenital distance as a marker for normal genital development. In humans, girls with CAH have been shown to have longer perineal lengths than their normal counterparts [18]. Hsieh et al demonstrated shorter anogenital distances in boys with genital anomalies (i.e. hypospadias and cryptorchidism), establishing a link between normal genital development and perineal length in humans [6].

Rodent studies have explored links between anogenital distance, penile length, and testicular development and function [12], [13], [15]. The current report established that AGD might also help determine genital development and function in adult men. While penile length, AGD, and testicular volume were all correlated, only testis size and AGD predicted sperm production in men. Interestingly, while significant differences in genital measurements were noted between men of different ethnicities, AGD remained associated with fatherhood across races. While ancestry may impact absolute genital lengths, it appears that perturbances in genital development can still be captured by measuring AGD.

Rodent studies have used AGD to show abnormal androgen function after exposure to chemicals known to disrupt androgen-mediated pathways [13], [19], [20]. Foster and colleagues reported that exposure of developing rats to di-n-butyl phthalate (DBP), an anti-androgen, led to reproductive tract anomalies, reduced anogenital distance, and impaired testosterone production [13].

Swann et al demonstrated that mothers exposed to higher levels of endocrine disruptors birthed sons with shorter perineal lengths, linking environmental exposure and human genital development [14]. Torres-Sanchez reported that boys born to mothers with higher early gestational levels of an organochlorine pesticide metabolite (DDE) had reduced anogenital lengths [9]. Interestingly, several rat studies have also established a critical masculinization programming window where endocrine disruptors can permanently alter genital development, growth, and function [15], [16]. While postnatal endocrine action was also found to be important, Welsh et al showed that gestational exposure can irreparably damage genital development [16]. If confirmed in humans, such studies suggest that gestational exposures may play a critical role in male fertility and support a fetal origin of the testicular dysgenesis syndrome. While the current report did not elicit a history of environmental toxins, as groups have postulated an environmental explanation to declining fertility, in utero exposures could be a partial explanation for abnormal male genital development and reduced AGD.

Certain limitations warrant mention. As a referral center for male infertility, it was not always possible to blind observers to the men's diagnoses or fatherhood status which theoretically can lead to observer bias. Although, the magnitude of observed differences in AGD between fathers and infertile men (i.e. 40% in mean AGD and 45% in median AGD) suggests that any bias would be unlikely to affect the overall conclusions. Moreover, the current method of AGD measurement in adult men has not been studied, thus its accuracy and reproducibility were difficult to assess other than the performed comparison of measurements between investigators. Future studies are necessary to compare techniques for measurement as well as other anatomic locations of the AGD measurement. In addition, only men referred to and evaluated in our clinic were eligible for enrollment; therefore, it is possible that our patient population does not represent all infertile men. It is also important to note that the fertile controls were significantly older than the infertile patients. While age was not associated with AGD after accounting for fatherhood status and no evidence of effect modification by age was found, it possible that AGD could change with age. In addition, while all patients were measured in the same position, some men were measured at the time of surgery under general anesthesia while others were awake. It is conceivable that anesthesia may affect measurements, although stratifying by anesthesia status did not affect the conclusions. Nevertheless, our study represents the first analysis of anogenital distance in adult men and demonstrated an association between perineal length and fertility status. As such, AGD may predict normal male genital development, and could therefore provide a novel metric to assess reproductive potential in men.
